# Supershear Rayleigh wave imaging for quantitative assessment of biomechanical properties of brain using air-coupled optical coherence elastography

**DOI:** 10.1063/5.0160213

**Published:** 2023-10-30

**Authors:** Yirui Zhu, Jiulin Shi, Tomas E. Gomez Alvarez-arenas, Chenxi Li, Haohao Wang, Hongling Cai, Dong Zhang, Xingdao He, Xiaoshan Wu

**Affiliations:** 1 School of Testing and Opto-electric Engineering, Nanchang Hangkong University, Nanchang 330063, China; 2 School of Physics, Nanjing University, Nanjing 210093, China; 3 Ultrasonic and Sensors Technologies Department, Information and Physical Technologies Institute, Spanish National Research Council, Serrano 144, 28006 Madrid, Spain

## Abstract

Recently, supershear Rayleigh waves (SRWs) have been proposed to characterize the biomechanical properties of soft tissues. The SRWs propagate along the surface of the medium, unlike surface Rayleigh waves, SRWs propagate faster than bulk shear waves. However, their behavior and application in biological tissues is still elusive. In brain tissue elastography, shear waves combined with magnetic resonance elastography or ultrasound elastography are generally used to quantify the shear modulus, but high spatial resolution elasticity assessment in 10 *μ*m scale is still improving. Here, we develop an air-coupled ultrasonic transducer for noncontact excitation of SRWs and Rayleigh waves in brain tissue, use optical coherent elastography (OCE) to detect, and reconstruct the SRW propagation process; in combing with a derived theoretical model of SRWs on a free boundary surface, we quantify the shear modulus of brain tissue with high spatial resolution. We first complete validation experiments using a homogeneous isotropic agar phantom, and the experimental results clearly show the SRW is 1.9649 times faster than the bulk shear waves. Furthermore, the propagation velocity of SRWs in both the frontal and parietal lobe regions of the brain is all 1.87 times faster than the bulk shear wave velocity. Finally, we evaluated the anisotropy in different brain regions, and the medulla oblongata region had the highest anisotropy index. Our study shows that the OCE system using the SRW model is a new potential approach for high-resolution assessment of the biomechanical properties of brain tissue.

## I. INTRODUCTION

The brain is the most advanced part of the nervous system; the brain controls the human mind and dominates all processes of human activity. Over the past two decades, elastography studies in the field of brain medicine, such as those involving magnetic resonance elastography (MRE), have demonstrated the great potential of detecting the biomechanical properties of brain tissue to diagnose, treat, and prevent brain tissue diseases.^1–3^ Several studies have shown that the stiffness of brain tissue is correlated with various clinical disorders; in addition, the stiffness of brain tissue decreases significantly with age, and a strong relationship between the viscoelastic properties of brain tissue and body behavior has been shown.^4–6^ Therefore, quantifying the biomechanical properties of brain tissue is essential for understanding brain tissue function and disease. However, brain tissue with more than 80% water content is a typical viscoelastic medium, and characterization of its biomechanical properties (anisotropy, viscoelasticity, etc.) requires consideration of complex boundary conditions and wave models. Due to the limited resolution of conventional MRE and the limitations of existing shear wave models, higher resolution techniques and more accurate mechanical wave models are needed to quantify the biomechanical properties of brain tissue.

**FIG. 1.**
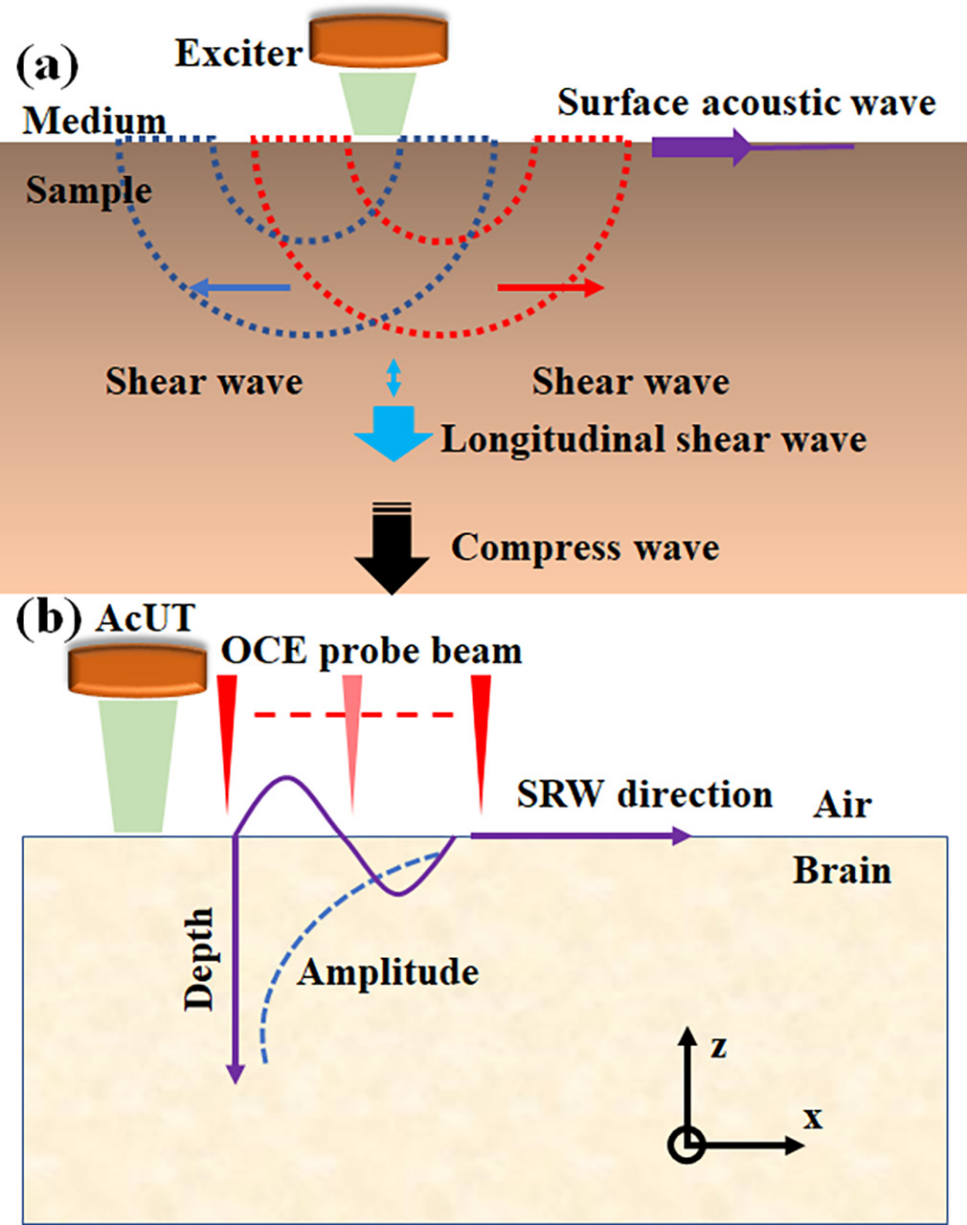
(a) Schematic diagram of the propagation of different mechanical wave modes in soft tissue. (b) Schematic diagram of SRW propagation in brain tissue after acoustic radiation force excitation.

By combining different mechanical wave models and detection modalities, various dynamic elastography techniques have been developed and widely used to assess the biomechanical properties of biological tissues. For example, with the development of ultrasonic transducer technology,^7,8^ shear wave-based ultrasound elastography (USE) has been applied in the diagnosis and treatment of clinical diseases (liver cirrhosis, breast cancer, and ocular tissue disease).^9–12^ Recently, wave-based dynamic optical coherence elastography (OCE) techniques have rapidly developed, with the advantages of nondestructive, micron-level spatial resolution.^13–15^ When an external excitation source acts on the surface of an elastic medium in a contact or noncontact manner, a mechanical disturbance is generated and spreads or propagates from the excitation location, generating a mechanical wave. The type and propagation speed of the mechanical wave are determined by the mechanical properties and boundary conditions of the tissue, as shown in Fig. 1(a). Shear waves are also known as transversal waves (a type of bulk wave), longitudinal shear waves, and Rayleigh waves and have been widely used in the field of OCE research. Shear wave-based OCE was previously proposed to characterize the shear modulus of biological tissues, because the propagation velocity of shear waves depends directly on the mechanical properties of the medium.^16,17^ Longitudinal shear waves have both transverse and longitudinal motion components and propagate in the axial direction of the OCE system at the velocity of the transverse waves; therefore, these waves can be detected by the OCE system.^18–20^ Rayleigh waves are typical surface acoustic waves (SAWs) that have transverse and longitudinal motion components and are guided by the surface of the tissue to propagate along the surface. The amplitudes of Rayleigh waves decay exponentially in the depth direction, and the waves propagate to a depth of approximately one wavelength. Therefore, Rayleigh wave-based OCE is widely used to detect the biomechanical properties of biological tissues such as agar, ocular tissue, and skin tissue, as the limitation of the imaging depth is not an issue.^21–23^ However, simple bulk transverse waves and Rayleigh waves cannot be used to accurately quantify the biomechanical properties of biological tissues with complex boundary conditions. Considering the boundary characteristics of biological tissues and the effect of viscoelastic properties on the dispersion of mechanical waves, more complex mechanical wave models, such as the Rayleigh–Lamb wave model, are needed to analyze the biomechanical properties of the cornea, retina, and skin.^21,24^ However, quantifying the elastic moduli of biological tissues by analyzing wave dispersion requires complex computational procedures, as the medium thickness and wave bandwidth tend to introduce false estimates into the detection results.^25^

Several previous studies have used wave-based OCE techniques to assess the biomechanical properties of brain tissue. A longitudinal shear wave model combined with compression OCE was used to assess the two-dimensional elasticity of an *ex vivo* mouse brain.^19^ Reverberant shear wave field-based OCE was used to preliminarily estimate the shear wave speed in the brains of recently deceased mice.^26^ Air-pulse-based OCE was used to assess the differences in the biomechanical properties of different regions of *ex vivo* brain tissue, and the hippocampus was found to be softer than the cerebral cortex.^27^ Acoustic radiation force-based OCE was used for *ex vivo* investigations of the mouse cerebral cortex and stroke rat model cerebral cortex, and the results showed that the system can distinguish the elasticity of different cortex samples with better spatial resolution than previous approaches.^28^ However, preliminary studies of wave-based OCE techniques in brain tissue did not consider boundary conditions. Considering that OCE images in brain tissue are acquired at a depth of 1–3 mm, the free surface boundary conditions allow SAWs to be used to quantify the biomechanical properties of brain tissue. Recently, a surface wave with a velocity between that of bulk longitudinal and transverse waves was demonstrated in theory and experiments and used for elastic characterization of soft tissues. Unlike conventional Rayleigh waves, which have lower velocities than bulk transverse waves, these surface waves have velocities that are approximately twice as fast as body transverse waves; these waves propagate along the surface of the medium and have been termed supershear Rayleigh waves,^29^ pseudo-P waves,^30^ leaky Rayleigh waves,^31^ leaky surface waves,^32^ nongeometric PS waves,^33^ and supershear evanescent waves^34^ in previous literature reports. Several previous papers have used this surface wave-based OCE technique to evaluate the biomechanical properties of phantoms, *ex vivo* human corneas, and human skin tissue, demonstrating the feasibility of this approach.^34–36^

In the present, the supershear Rayleigh waves (SRWs) are proposed to characterize the behavior of elastic waves in brain tissue. The theoretical derivation of the SRW is developed and demonstrates that the propagation direction is along the brain tissue surface and that the SRW velocity is considerably faster than the bulk transverse wave velocity. An air-coupled ultrasound transducer (AcUT) is used to excite the sample to produce mechanical vibrations, and a phase-resolved optical coherence tomography (OCT) system is used to detect the SRW signal. We first perform validation experiments using a homogenous phantom model, obtaining SRW propagation results in *ex vivo* porcine brain tissue; then, we obtain the three-dimensional (3D) surface reconstruction (x, z, t) results of the SRWs. To the best of our knowledge, this is the first time that noncontact OCE has been used to assess SRW behavior in brain tissue. The results suggest that SRW-based OCE technology can be used in the future for noncontact, high spatial resolution quantitative evaluation of the biomechanical properties of brain tissue.

## II. RESULTS

### A. Phantom experiment results

**FIG. 2.**
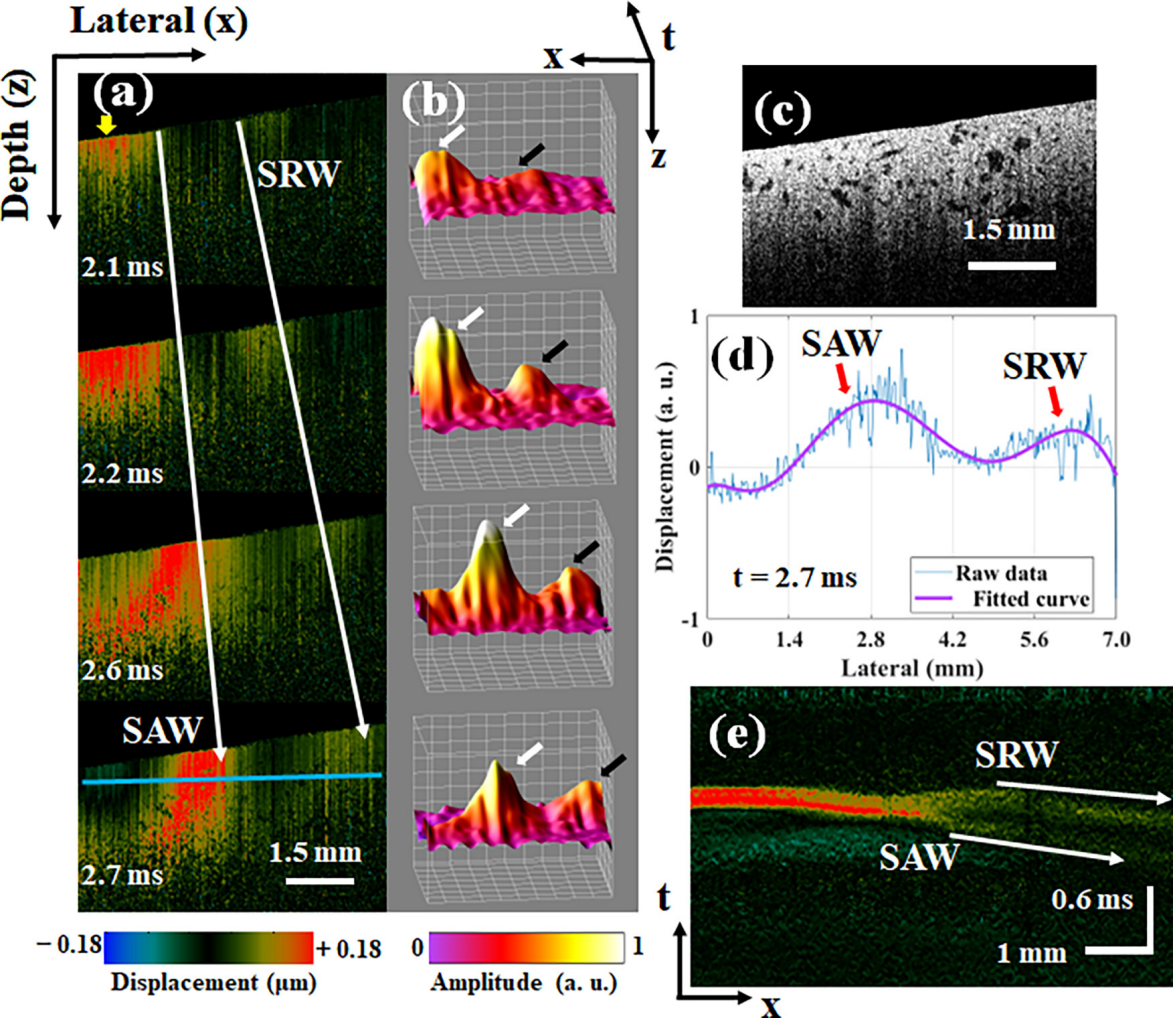
The AcUT-OCE results of the SAW and SRW propagation processes in the phantom. (a) 2D vibration displacement propagation images of the SAW and SRW in the phantom at different times. The yellow arrow in the top image indicates the excitation point. (b) 3D surface reconstruction (x, z, t) of the SAW and SRW; the white arrow indicates the SAW, and the black arrow indicates the SRW. (c) The 2D structure of phantom. (d) The displacement profiles of the SRW and SAW at the depth marked by the blue line in the bottom slice of Fig. 2(a) at 2.7 ms. (e) The spatiotemporal displacement maps of the SRW and SAW at the depth marked by the blue line in the bottom slice of Fig. 2(a).

We first completed phantom experiments to verify the feasibility of the proposed AcUT-OCE system and to analyze the SRW behaviors in a homogeneous medium. The 2D (x, t) wavefield propagation images of SAWs and SRWs in the phantom at different times are shown in Fig. 2(a), and the corresponding 2D structure of phantom is shown in Fig. 2(c). After the acoustic radiation force excitation, the vibration displacement propagates from the excitation position to the surrounding medium, generating an elastic wave, as shown in the top slice in Fig. 2(a), and the yellow arrow indicates the location of the acoustic radiation force excitation. The wavefronts of the SRW and SAW can be clearly observed after 2.1 ms, and the propagation speed of the SRW is faster than that of the SAW, approximately twice as fast as that of the bulk shear wave, and decays faster due to leakage into the bulk. Moreover, the wavefield behavior of the SRW and SAW can be clearly observed in the 3D (x, z, t) surface reconstruction results, as shown in Fig. 2(b). The white arrow indicates the SAW, and the black arrow indicates the SRW. Compared to the SAW, the SRW travels farther in the same amount of time but has a lower amplitude. The 3D (x, z, t) reconstruction results of the SRW and SAW propagation processes are shown in supplementary material video 1. Then, the displacement profiles of the SAW and SRW were obtained at the depth marked by the blue line in the bottom slice of Fig. 2(a) at 2.7 ms, as shown in Fig. 2(d). Along the depth direction, the 3D (x, z, t) dataset is resliced to obtain the spatiotemporal displacement maps of the SRW and SAW at the depth marked by the blue line in the bottom slice of Fig. 2(a), as shown in Fig. 2(e).

Near the excitation point, it is difficult to distinguish the SRW and SAW modes, while at 3–4 mm from the excitation point, the SRW clearly propagates faster than the SAW, and the amplitude decays as the wave propagates [see Fig. 2(e)]. The time contours of the SRW and SAW can be clearly observed [see Fig. 2(e)], and the wave speed can be obtained by calculating the reciprocal of the slope of the local maxima of the SRW and SAW, i.e., by calculating the propagation distances of the elastic waves during the different detection times in Fig. 2(e). At the same time, we note that the SNR of SRW is lower than that of SAW, which is due to the fact that SRW is leaky and its energy decays rapidly during propagation. Each experiment was repeated three times, and the mean and variance of the mechanical wave velocity were calculated. The speeds of the SRW and SAW were 5.60 ± 0.22 and 2.72 ± 0.11 m/s, respectively. For a nearly incompressible semi-infinite medium, according to Eq. (17), the ratio of the Rayleigh wave and shear wave speeds is^34^ $c_{R}/c_{s}\approx 0.9553$; therefore, we calculated the shear wave speed in the phantom as 2.85 ± 0.12 m/s (which is an approximately semi-infinite medium). Then, the ratio of the SRW and shear wave speeds is calculated as $c_{\mathrm{SRW}}/c_{s}\approx 1.9649$, which shows that the speed of the SRW in the phantom is approximately twice the speed of the bulk transverse wave. This result is consistent with a previous report and theoretical calculations.^34,35^

### B. *Ex vivo* porcine brain tissue experiment results

**FIG. 3.**
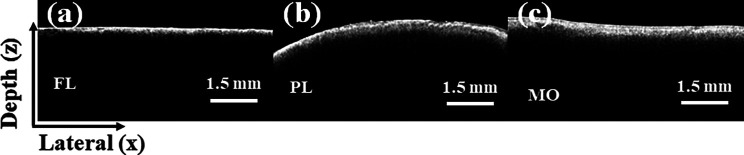
The 2D OCT structure results of different regions of brain tissue. (a) The frontal lobe (FL) region. (b) The parietal lobe (PL) region. (c) The medulla oblongata (MO) region.

**FIG. 4.**
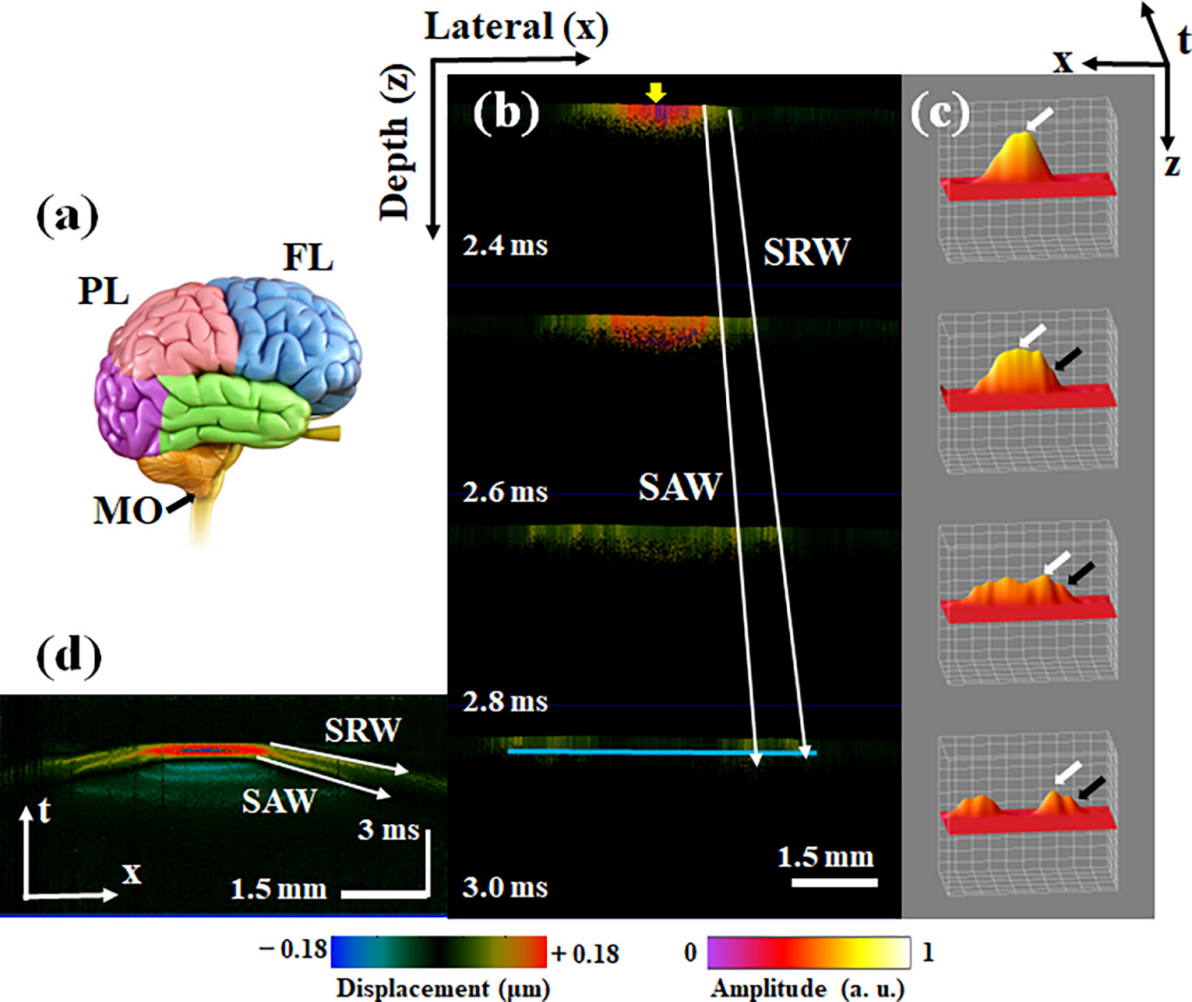
The AcUT-OCE results of the SRW and SAW propagation processes in the FL. (a) The different detection regions in *ex vivo* brain tissue. (b) The SRW and SAW propagation processes at different times, the yellow arrow indicates the location of acoustic radiation force excitation. (c) 3D (x, z, t) surface reconstruction results, with the white and black arrows indicating the SAW and SRW, respectively. (d) Spatiotemporal image of SRW and SAW propagation, and the white arrow indicates the time contours of the SRW and SAW.

To explore SRW behavior in brain tissue, we completed *ex vivo* OCE experiments in porcine brains and determined the relationship between the SRW speed and shear wave speed. As shown in Fig. 3, we first obtained the 2D OCT structure results of frontal lobe (FL), parietal lobe (PL), and medulla oblongata (MO) of the porcine brain *ex vivo*. Figure 4(a) gives the different locations of FL, PL, and MO regions in the brain tissue structure. In the FL, prior to 2.4 ms, the SRW and SAW cannot be clearly distinguished in the middle approximately 4 mm from the excitation region, as shown in the top plots in Figs. 4(b) and 4(c). After 2.6 ms, the SRW wavefront propagates farther than the SAW wavefront at the same detection moment due to its faster propagation speed, as shown in Fig. 4(b). The 3D (x, z, t) surface reconstruction results at the corresponding moment are presented in Fig. 4(c), and the propagation of the SRW and SAW can be clearly observed. The white and black arrows indicate the SAW and SRW wavefronts, respectively. The full videos of the 2D and 3D reconstructed SRW and SAW propagation processes in the FL are provided in supplementary material videos 2 and 3, respectively. Furthermore, the 3D dataset containing the M-B scans is resliced along the depth direction to obtain spatiotemporal displacement maps, and the results show the clear propagation time contours of the SRW and SAW in the FL, as shown in Fig. 4(d). The velocities of the SRW and SAW in the FL were calculated as 2.31 ± 0.25 and 1.18 ± 0.02 m/s, respectively. The speed of the shear wave was calculated as 1.23 ± 0.05 m/s according to the SAW speed. The ratio of the SRW and shear wave speeds is calculated as $c_{\mathrm{SRW}}/c_{s}\approx 1.87$.

**FIG. 5.**
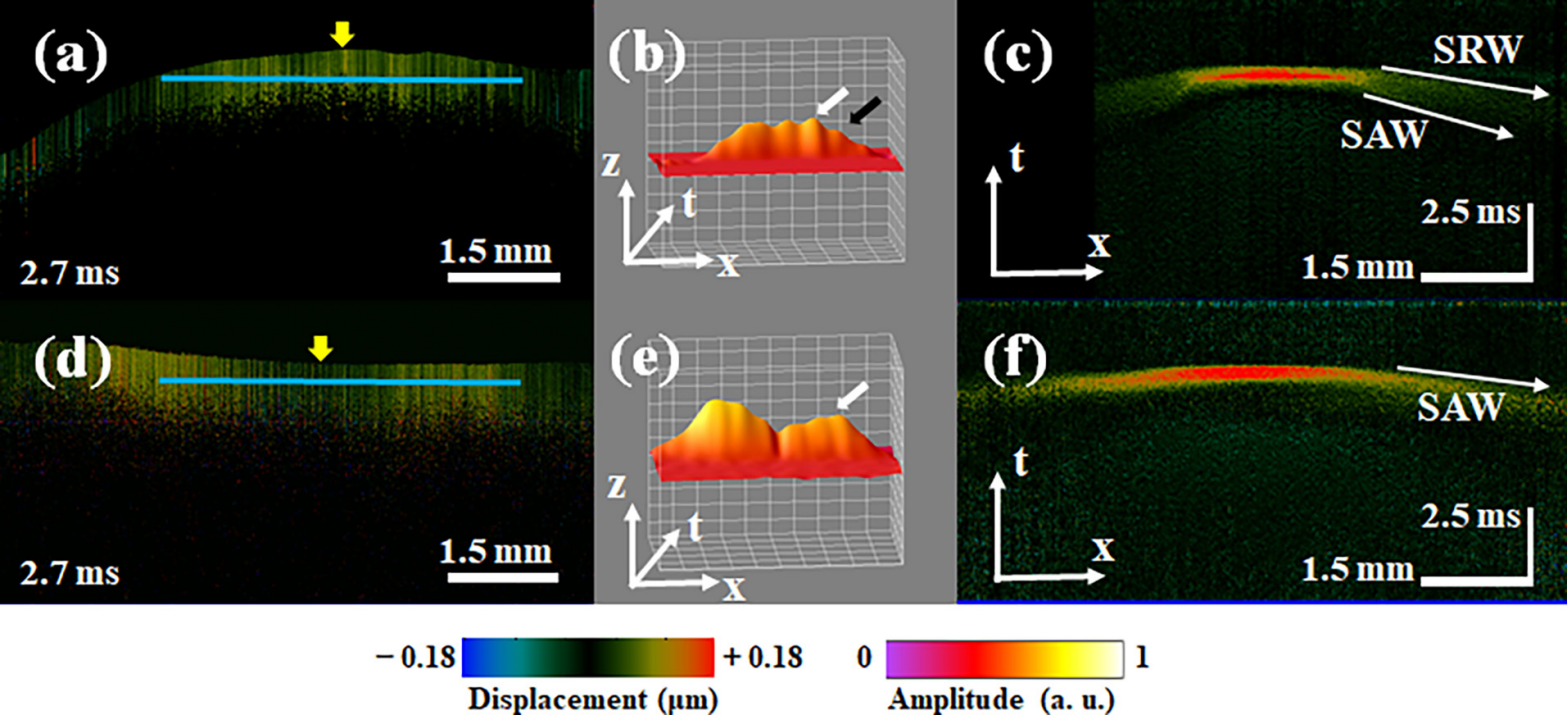
The AcUT-OCE results of the SRW and SAW propagation processes in the PL and MO. (a) Vibration displacement image of SRW and SAW propagation in the PL at 2.7 ms, the yellow arrow indicates the location of acoustic radiation force excitation. (b) 3D surface reconstruction results of the SRW and SAW at 2.7 ms; the white and black arrows indicate the SAW and SRW, respectively. (c) Spatiotemporal image of SRW and SAW propagation in the PL. (d) Vibration displacement image of SAW propagation in the MO at 2.7 ms, the yellow arrow indicates the location of acoustic radiation force excitation. (e) 3D surface reconstruction result of SAW at 2.7 ms; the white arrow indicates the SAW. (f) Spatiotemporal image of SAW propagation in the MO.

In the PL, the vibration displacement image of the SRW and SAW propagation processes at 2.7 ms is shown in Fig. 5(a), and there is no significant difference between the SRW and SAW propagation processes. However, the 3D (x, z, t) surface reconstruction results show that the wavefront of the SRW propagates significantly farther than that of the SAW. The full videos of the 2D and 3D reconstructed SRW and SAW propagation process are shown in supplementary material videos 4 and 5. The spatiotemporal displacement maps of the SRW and SAW propagation processes were obtained at the depths marked by the solid blue lines in Fig. 5(a), as shown in Fig. 5(c). The speeds of the SRW and SAW were calculated as 2.52 ± 0.27 and 1.29 ± 0.35 m/s, respectively. Furthermore, the speed of the shear wave in the PL was calculated as 1.35 ± 0.36 m/s according to the SAW speed. The ratio of the SRW and shear wave speeds was calculated as $c_{\mathrm{SRW}}/c_{s}\approx 1.87$. In the MO, the vibration displacement image of elastic propagation at 2.7 ms is shown in Fig. 5(d); however, during 10 s of imaging, no significant propagation of the SRW was observed, and only propagation of the SAW was detected. Figure 5(e) illustrates that only the SAW propagates after the acoustic radiation force excites the MO. The spatiotemporal displacement map in Fig. 5(f) has only one time profile, suggesting that only the SAW mode was detected in the MO region. The speeds of the SAW and shear wave in the MO were calculated as 4.09 ± 0.66 and 4.28 ± 0.69 m/s, respectively.

### C. Experimental results of anisotropy in the FL, PL, and MO

**FIG. 6.**
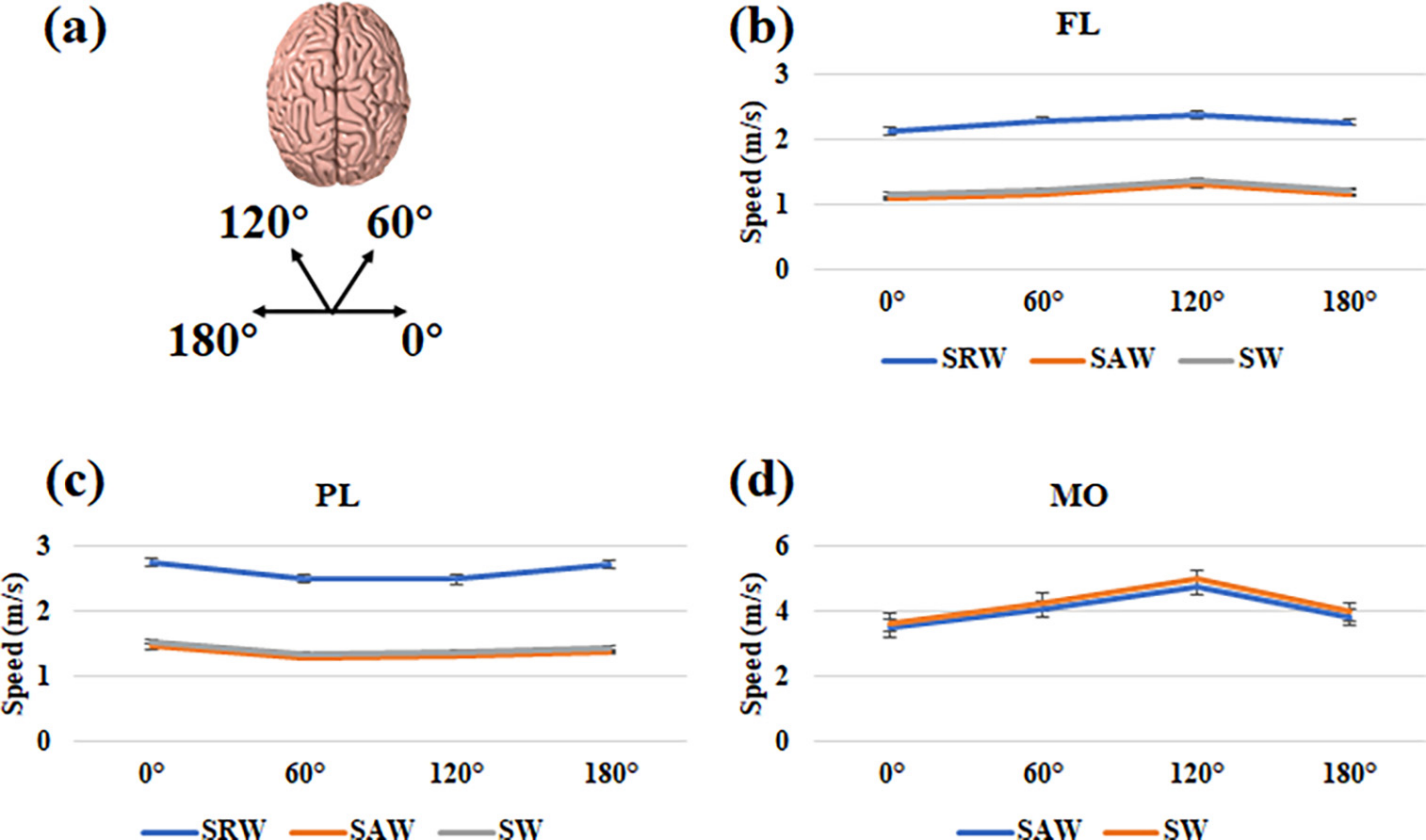
The results in the FL, PL, and MO. (a) Experimental protocol in the FL, PL, and MO regions; elastic wave data were acquired in four directions: 0°, 60°, 120°, and 180°. (b) The shear modulus results in different directions in the FL region. (c) The shear modulus results in different directions in the PL region. (d) The shear modulus results in different directions in the MO region.

**FIG. 7.**
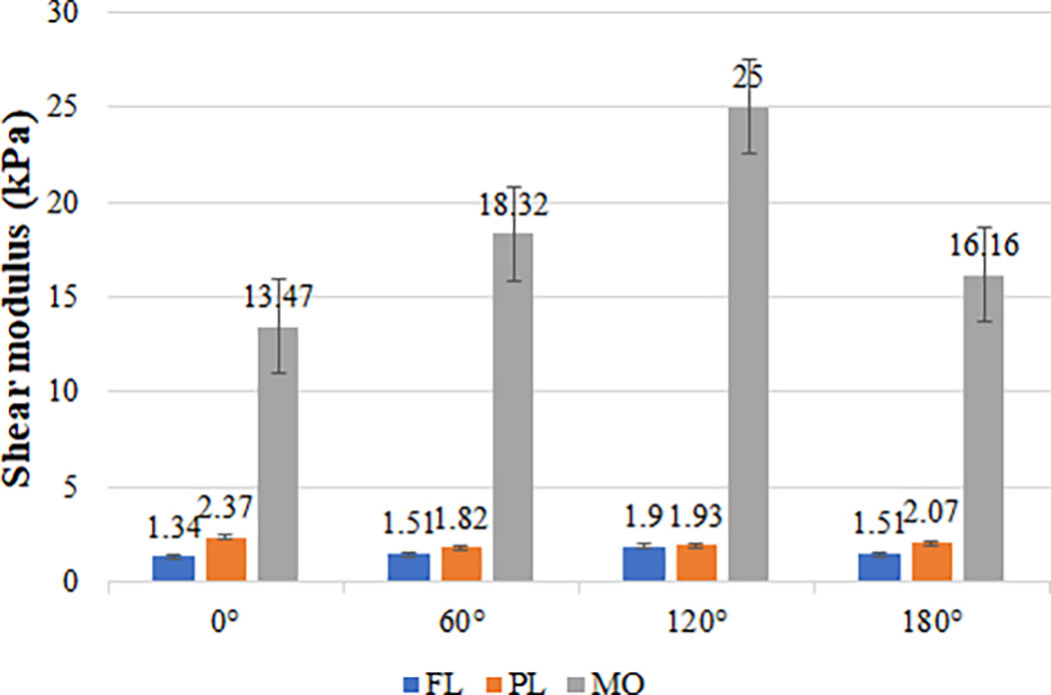
Detailed calculations of the average shear modulus in different brain regions, and the error bars indicate the standard deviation.

We designed a simple experimental protocol to investigate the anisotropy of the biomechanical properties of brain tissue based on the OCE results. As shown in Fig. 6(a), elastic wave data were acquired in the FL, PL, and MO regions in the brain in four directions: 0°, 60°, 120°, and 180°.^35^ This study used index ξ, which is defined as $\xi =(\mu_{\text{max}}-\mu_{\text{min}})/{(\mu_{\text{max}}+\mu }_{\text{min}})$, to characterize the anisotropy of different regions of brain tissue; here, $\mu_{\text{max}}$ and $\mu_{\text{min}}$ denote the maximum and minimum values of the shear modulus, respectively. According to Eq. (8), to calculate μ, if the sample is an isotropic medium, ξ should be equal to 0. During the experiment, the average value of the mechanical wave propagation velocity was calculated after three experimental data were collected for each sample. A t-test was taken to assess the differences between the shear moduli of different brain tissues. In the FL and PL regions, the SAW and SRW results were acquired simultaneously in each direction, as shown in Figs. 6(b) and 6(c). In the FL region, the maximum value of the elastic wave speed was obtained along the 120° direction; however, in the PL region, the maximum value of the elastic wave speed was obtained along the 0° direction. ξ values of 0.17 and 0.13 were calculated for the FL and PL, respectively. In the MO region, only the SAW was observed in the OCE experimental results, with no SRWs, and the maximum elastic wave velocity was obtained in the 120° direction, as shown in Fig. 6(d). The calculated ξ value for the MO region was 0.40, which is larger than the values calculated for the FL and PL regions. According to Eq. (18), the detailed results of the calculations of the average shear modulus in different brain regions are shown in Fig. 7, and this represents the mean and standard deviation of the shear modulus obtained by repeating the test three times along different directions in different regions of the brain tissue.

## III. DISCUSSION

In this work, we explored the behavior of supershear Rayleigh waves (SRWs) in brain tissue for the first time; these waves propagate along the surface at velocities faster than bulk shear waves. We derived a theoretical model for the supershear Rayleigh wave and presented the general solution of the Rayleigh equation. Furthermore, we developed an air-coupled ultrasound transducer-based optical coherent elastography (AcUT-OCE) system for nondestructive, noncontact excitation, and detection of elastic wave propagation in brain tissue. A homogeneous, isotropic agar phantom was used in the OCE experiments, and the behavior of the supershear Rayleigh waves was observed. Furthermore, the OCE results in the FL and PL regions of *ex vivo* porcine brain tissue showed the propagation of both SRWs and SAWs, while in the MO region, only SAW propagation was observed. Finally, we defined the index ξ, which was used to evaluate the anisotropy of biomechanical properties in different regions of brain tissue.

MRE using a shear wave model can be used to obtain direct quantitative measurements of the shear modulus and shear viscosity in brain tissue; as a result, this technology has been widely used for noninvasive stiffness assessment of brain tissue.^37^ The altered biomechanical properties of the brain tissue mainly affect the wavelength of the shear waves. In terms of the shear wave behavior during MRE, when global vibration is applied, a low-amplitude shear wave is generated that propagates from the surface of the meninges toward the center of the brain.^38^ The increase in the stiffness of the brain tissue leads to an increase in the shear wave velocity and therefore the wavelength. However, brain tissue has a complex histological structure and boundary conditions, and the behavior of elastic waves, reflections, interferences, dispersions, etc., creates complex wave fields and more modes. Therefore, in addition to improving the MRE resolution to enhance the accuracy of biomechanical property assessment in brain tissue, it is equally important to analyze the complex behavior of elastic waves in brain tissue to accurately quantify the stiffness of brain tissue. In this work, we investigated the behavior of SRWs in brain tissue using an AcUT-OCE system. Although it is difficult for OCE to penetrate the skull for direct imaging of soft tissues due to the thickness of the skull, applying the OCE technique, which has advantages such as high spatial resolution and high signal-to-noise ratio, through an optical window to investigate brain tissue is an important approach for assessing brain tissue stiffness. Moreover, we developed an air-coupled ultrasound transducer with a center frequency of 250 kHz, which avoids the use of couplants and allows direct noncontact excitation of elastic waves in brain tissue in air.

We first used an isotropic homogeneous agar phantom to verify the behavior of the SRWs and the feasibility of the AcUT-OCE system. We detected SRWs propagating along the surface approximately 3 mm from the excitation region, and the wavefronts of the SRW and SAW were clearly observed in the 3D (x, z, t) surface reconstruction results [Fig. 2(b)]. Furthermore, the SRW velocity was approximately 1.9649 faster than the bulk shear wave velocity. The theoretical value of the SRW speed in a semi-infinite elastic medium was estimated to be between 1.9554 and 2.9049 times the bulk shear wave velocity in previous reports, which is consistent with our experimental results.^34,35^ In the FL, the propagation processes of the SRW and SAW were clearly detected by the AcUT-OCE system after 2.6 ms, and the wavefront of the SRW propagated farther than the wavefront of the SAW with a lower amplitude and a speed of 2.31 ± 0.25 m/s, as shown in Figs. 4(b) and 4(c). Although the relationship between the SRW velocity and bulk shear wave velocity in biological tissues has not been accurately calculated, we provide an estimate based on our experimental results, which is approximately $c_{\mathrm{SRW}}/c_{s}\approx 1.87$. Compared to the theoretical value of 1.9554, the error is approximately 4.37%. Similarly, in the PL, we analyzed the behaviors of the SRW and SAW, calculated the velocity of the SRW to be approximately 2.52 ± 0.27 m/s [Figs. 5(b) and 5(c)], and estimated the ratio of the SRW and shear wave velocities in the PL to be $c_{\mathrm{SRW}}/c_{s}\approx 1.87$. Interestingly, this result is the same as that in the FL, which demonstrates the stability of the SAW and shear wave velocities in brain tissue assessed using the OCE system. However, in the MO, we detected only SAWs and no SRWs, which we believe may be due to the fast SRW velocity in the MO region and the small area scanned by the AcUT-OCE system.^35^

Furthermore, we discussed the shear modulus in different regions of brain tissue, which is an important research area in elastography studies of the brain. Kruse *et al.* used MRE to assess the shear stiffness of the gray and white matter in the human brain and showed that the shear stiffness of white matter was significantly higher than that of gray matter.^38^ Johnson *et al.* assessed the shear stiffness of the subcortical gray matter and the surrounding white matter and found that the gray matter was stiffer than the surrounding white matter.^39^ However, Guo *et al.* reached a different conclusion, observing that the gray matter in these regions was softer than the surrounding tissue.^40^ In addition, the difference between the stiffness of brain tumors, such as gliomas, meningiomas, pituitary tumors, and adenomas, and the stiffness of the surrounding healthy tissue has been the focus of research.^41–44^ In our study, we precisely calculated the shear modulus of different brain regions based on AcUT-OCE results and discussed the anisotropy in the results. As shown in Fig. 7, the PL region has the lowest shear modulus among the investigated brain regions, while the MO region has a significantly higher shear modulus. Furthermore, we defined the index ξ to characterize the anisotropy of the brain tissue, and the results showed that the ξ in the MO region was 0.40, which was significantly higher than that in the FL (0.17) and PL (0.13). We believe that this result is related to the tissue structure of the MO, which is composed of a large number of fibers in the corticospinal tract, such as muscle fibers, as the shear wave propagates faster along the fiber direction.^45,46^ Notably, the effect of brain tissue anisotropy on elasticity assessment has been an important research direction, and many researchers have worked on optimizing algorithms for the reconstruction of the elastic modulus in anisotropic brain tissue.^47^ Our experimental study may provide a new approach.

Most previous MRE studies were based on harmonic motion, where excitations at specific frequencies were applied to brain tissue and the displacement field was imaged.^48^ However, the viscoelastic behavior of brain tissue suggests that its shear biomechanical properties should be a complex modulus with real and imaginary parts, representing elastic and viscous behavior, respectively.^49^ The assessment of the shear modulus with MRE technology can be used to characterize only the macroscopic properties of brain tissue at the wavelength scale, and the loss modulus is closely related to the number of neurons with more cellular structures and geometries; moreover, the MRE resolution is not sufficient for measurements in the cerebral cortex.^50,51^ The results of our AcUT-OCE experiments show that accurate and stable elasticity estimation can be accomplished by detecting SRWs in brain tissue, especially in cortical regions with complex tissue structures. First, after AcUT excitation of SRWs in brain tissue, OCT is used to quantify the shear modulus of the brain tissue at micrometer-level spatial resolution, and the velocity of the shear wave is calculated directly based on the velocity of the SRW, regardless of the thickness and geometry the different brain regions. Second, it has been shown that the SRW velocity is negatively correlated with pressure,^35^ which differs from the typical Rayleigh wave; thus, by evaluating the SRW velocity, we can discuss some issues related to intracerebral pressure and cerebrospinal fluid pressure without considering that the SRW velocity is too fast to be accurately detected by the OCE system. In addition, the SRW velocity is sensitive to the anisotropy of the medium,^35^ which is a key issue to be addressed in future MRE studies, and the OCE results are more accurate for assessing the anisotropy of brain tissue.

However, there are some limitations in this prospective study that should be considered. First, a further experimental AcUT-OCE study with *in vivo* brain tissue should be performed, which may indicate physiological conditions such as the intracerebral pressure as well as the boundary conditions determined by the cerebrospinal fluid environment may alter the behavior of elastic waves in brain tissue and, thus, affect the elasticity assessment results. In fact, for *in vivo* brain tissue elastography, we need to design more rigorous experimental protocols and use more complex physical models to quantify the elastic modulus. First, if we design and fabricate a cranial window to accomplish elastography of brain tissue, we can still quantify the elastic modulus using the SRW model with free surface boundary conditions in the absence of cranial bone in the cranial window region. However, if we adopt the tissue transparency technique to fabricate an *in vivo* transparent cranial window, then we need to consider the boundary conditions of the brain tissue in the case of cranial stress. This is beyond the scope of this article, and we will address it in the future work. Second, an AcUT with a center frequency of 250 kHz excites elastic waves at multiple frequencies, and in this study, we evaluated only the shear modulus (storage modulus) without discussing the energy loss associated with absorption and scattering in brain tissue, i.e., the loss modulus, due to our primarily prospective discussion of the behavior of SRWs in brain tissue. We will use Fourier analysis in future studies to discuss the viscoelasticity of brain tissue. Finally, the imaging depth of the OCE system is approximately 1–3 mm; thus, we only here focus on cortical regions with complex multilayer structures, and a more accurate depth-resolved elasticity assessment is needed. For the further work, the viscoelastic behavior of brain tissue and develop more accurate algorithms for depth-resolved elasticity quantification are to be studied.

## IV. CONCLUSION

SRW has been shown to be sensitive to material anisotropy, and SRW exhibits different behaviors for loading of material prestresses, which implies that SRW can also be used in an OCE approach to discuss the mechanical properties of biological tissues. In this work, we developed an optical coherent elastography system based on an air-coupled ultrasound transducer to investigate the behavior of supershear Rayleigh waves in brain tissue. In the FL and PL regions in the brain, we detected SRWs propagating along the surface with a velocity approximately 1.87 times the velocity of the bulk shear waves. We defined the index ξ to represent the anisotropy of different regions of brain tissue, and the results showed that the MO had the highest anisotropy index among the investigated brain regions. Our study provides a new perspective on elasticity assessments of brain tissue with free surface conditions that is sensitive to the anisotropy of brain tissue that involves directly calculating the shear modulus of brain tissue in micrometer-level based on the supershear Rayleigh wave velocity.

## V. METHODS

### A. The supershear Rayleigh wave model

As shown in Fig. 1(b), in the Cartesian coordinate system, the SRW is found in the *x–z* plane, the propagation direction is along the *x*-axis, if the direction of the acoustic radiation force produced by the AcUT is along the *z*-axis. When the acoustic radiation force is used to excite the sample, Hooke's law can be used to express the stress and strain relationship in the sample

$$\sigma_{i,\;j}=\delta_{i,\;j}\lambda \varepsilon_{i,\;j}+2\mu \varepsilon_{i,\;j},$$ (1)

where $\sigma_{i,\;j}$ and $\varepsilon_{i,\;j}$ are the strain and stress, respectively, λ and μ are Lame constants, and μ is the shear modulus. Here, the indices *i* and *j* take the values of the coordinate axes *x*, *y*, and *z*, and δ=1 if i=j and δ=0 if i ≠ j. The longitudinal elasticity modulus M is formulated as follows:^52^

$$M=\frac{\mu \left(3\lambda +2\mu \right)}{\lambda +\mu }.$$ (2)

We can then obtain another important elastic constant, i.e., Poisson's ratio ν, which is defined as the ratio of compression to tension in a medium under stress, as follows:

$$\nu =\frac{\lambda }{2\left(\lambda +\mu \right)}.$$ (3)

According to Newton's second law, we obtain Eq. (1) for the *x-*axis as follows:

$$\rho \frac{\partial^{2}u_{x}}{\partial t^{2}}-\left(\lambda +\mu \right)\frac{\partial \varepsilon }{\partial_{x}}-\mu \nabla^{2}u_{x}=0,$$ (4)

where ρ is the medium density, $u_{x}$ is the vibration displacement, and $\nabla^{2}$ is the Laplace operator. Equation (4) can be written for all coordinates using vector analysis as follows:

$$\rho \frac{\partial^{2}\vec{u}}{\partial t^{2}}=\left(\lambda +\mu \right)g\text{rad}\;\text{div}\;\vec{u}+\mu \nabla^{2}u.$$ (5)

Equation (5) shows that in an infinite solid elastic medium, two types of waves propagate with different speeds.^53^ We introduce the potential function φ and stream function ψ to characterize the superposition of longitudinal waves and transverse waves in the vector field of Eq. (5) as follows:

$$\vec{u}=\vec{u_{L}}+\vec{u_{T}}=\text{grad}\;\varphi +\text{rot}\;\vec{\psi }.$$ (6)

Considering $\text{rot}\;u_{L}=\text{div}\;u_{T}=0$, substituting Eq. (6) into Eq. (5), we obtain

$$\begin{array}{c} \frac{\partial^{2}\vec{u_{L}}}{\partial t^{2}}=c_{L}^{2}\nabla^{2}\vec{u_{L}},\\ c_{L}=\sqrt{\left(\lambda +\mu \right)/\rho }, \end{array}$$ (7)

$$\begin{array}{c} \frac{\partial^{2}\vec{u_{T}}}{\partial t^{2}}=c_{T}^{2}\nabla^{2}\vec{u_{T}},\\ c_{T}=\sqrt{\mu /\rho ,} \end{array}$$ (8)

where $c_{L}$ and $c_{T}$ are the longitudinal wave velocity and transverse wave (shear wave) velocity, respectively. Then, we assumed that the SRW is found within the *x–z* plane, the potential function φ and stream function ψ can be used to decouple $u_{L}$ and $u_{T}$ as follows:^54^

$$\begin{array}{c} \frac{1}{c_{L}^{2}}\frac{\partial^{2}\varphi }{\partial t^{2}}=\frac{\partial^{2}\varphi }{\partial x^{2}}+\frac{\partial^{2}\varphi }{\partial z^{2},}\\ \frac{1}{c_{T}^{2}}\frac{\partial^{2}\psi }{\partial t^{2}}=\frac{\partial^{2}\psi }{\partial x^{2}}+\frac{\partial^{2}\psi }{\partial z^{2}.} \end{array}$$ (9)

The harmonic solutions of Eq. (9) can be expressed as

$$\begin{array}{c} \varphi =A\;\text{exp}\left[i\left(\omega t-\beta x-\gamma_{L}z\right)\right],\\ \psi =-i\mathrm{Bexp}\left[i\left(\omega t-\beta x-\gamma_{T}z\right)\right], \end{array}$$ (10)

where A and B are constants and the wavenumbers $\gamma_{L}$ and $\gamma_{T}$ are defined by Cheeke^55^

$$\begin{array}{c} \gamma_{L}^{2}=\beta^{2}-k_{L,}^{2}\\ \gamma_{T}^{2}=\beta^{2}-k_{T}^{2}, \end{array}$$ (11)

where $k_{L}$ and $k_{T}$ are the wavenumbers of the longitudinal waves and transverse waves, respectively, and $\beta =k_{SW}$ is the wavenumber of the surface wave traveling along the *x*-axis. For the OCE experiments performed in this work, the brain is a semi-infinite solid with a free surface, and the boundary conditions are that the tangential and normal stresses are zero on the surface, which can be expressed as

$$\begin{array}{c} \sigma_{zz}{|}_{z=0}=\lambda \left(\frac{\partial^{2}\varphi }{{\partial x}^{2}}+\frac{\partial^{2}\varphi }{{\partial z}^{2}}\right)+2\mu \left(\frac{\partial^{2}\varphi }{{\partial x}^{2}}-\frac{\partial^{2}\varphi }{\partial x\partial z}\right)=0,\\ \sigma_{xz}{|}_{z=0}=\mu \left(\frac{\partial^{2}\varphi }{{\partial x}^{2}}+2\frac{\partial^{2}\varphi }{\partial x\partial z}-\frac{\partial^{2}\varphi }{{\partial x}^{2}}\right)=0. \end{array}$$ (12)

A homogeneous linear equation with respect to the coefficients A and B, i.e., the Rayleigh equation, is obtained as^30,56^

$$\left(\beta^{2}-\gamma_{T}^{2}\right)+4\beta^{2}\gamma_{L}\gamma_{T}=0.$$ (13)

We introduce a polynomial form with the following definitions:

$$\begin{array}{c} \eta \equiv \frac{k_{T}}{\beta }=\frac{c_{SW}}{c_{T}},\\ \xi =\frac{k_{L}}{k_{T}}\frac{c_{T}}{c_{L}}. \end{array}$$ (14)

Then, we can rewrite Eq. (13) as

$$\eta^{6}-8\eta^{4}+8\left(3-2\xi^{2}\right)\eta^{2}-16\left(1-\xi^{2}\right)=0.$$ (15)

Brain tissue can be viewed as an incompressible medium with a Poisson's ratio of approximately 0.5; therefore, $c_{T}\ll c_{L}$, and Eq. (15) can be simplified as

$$\eta^{6}-8\eta^{4}+24\eta^{2}-16=0.$$ (16)

According to the above equation, the forward propagating roots are approximately

$$\begin{array}{c} \eta =\left\{\begin{array}{l} 0.9553\\ 1.9661-0.5672i\\ 1.9661+0.5672i. \end{array}\right. \end{array}$$ (17)

The first real root is the Rayleigh wave, or the surface acoustic wave (SAW), traveling along the surface at a velocity lower than the bulk transverse velocity. The second root is a supershear Rayleigh wave propagating along the surface, which is approximately twice as fast as the bulk transverse velocity.^30^ According to Eq. (8), the shear modulus of the material can be determined by

$$\mu =\rho \times c_{T}^{2}.$$ (18)

For the brain tissue, the density is about of 1000 kg/m^3^.

### B. Phantom preparation

Here, a soft tissue-mimicking phantom with a concentration of 0.6% w/v is constructed to verify the feasibility of the SRW-based OCE experiments. Granulated agar is dissolved in distilled water at 25 °C, and the solution is heated to 95 °C with stirring. After the heating was stopped, the agar solution is then cooled to 60 °C with continuous stirring, and then 0.6% v/v of the intralipid solution is mixed with the agar solution to increase light scattering. After stirring for 5 min, the final solution is stored in a refrigerator at 4 °C for solidification. A density of 1000 kg/m^3^ is used to calculate the shear modulus of the phantom.

### C. *Ex vivo* porcine brain preparation

Brain tissue is procured from euthanized (no more than 1 h) pigs and placed in a 2% agarose gel solution of artificial cerebrospinal fluid, which reduces the effect of dried brain tissue on the experimental results. To evaluate the differences in the biomechanical properties of different regions of brain tissue, we performed OCE experiments on the frontal lobe (FL), parietal lobe (PL), and medulla oblongata (MO) of brain tissue. The air-coupled ultrasound transducer excites the brain tissue without a coupling agent, so artificial cerebrospinal fluid is extracted with a syringe to ensure that the brain tissue remained fresh during the OCE experiments. The brain tissue is immersed in artificial cerebrospinal fluid for preservation at the end of the experiment before completing the mechanical stretch test. The density of brain tissue is generally considered to be 1000 kg/m^3^, and this value is also used to calculate the shear modulus.^38^

### D. Air-coupled ultrasound transducer-based optical coherence elastography (AcUT-OCE) system setup

**FIG. 8.**
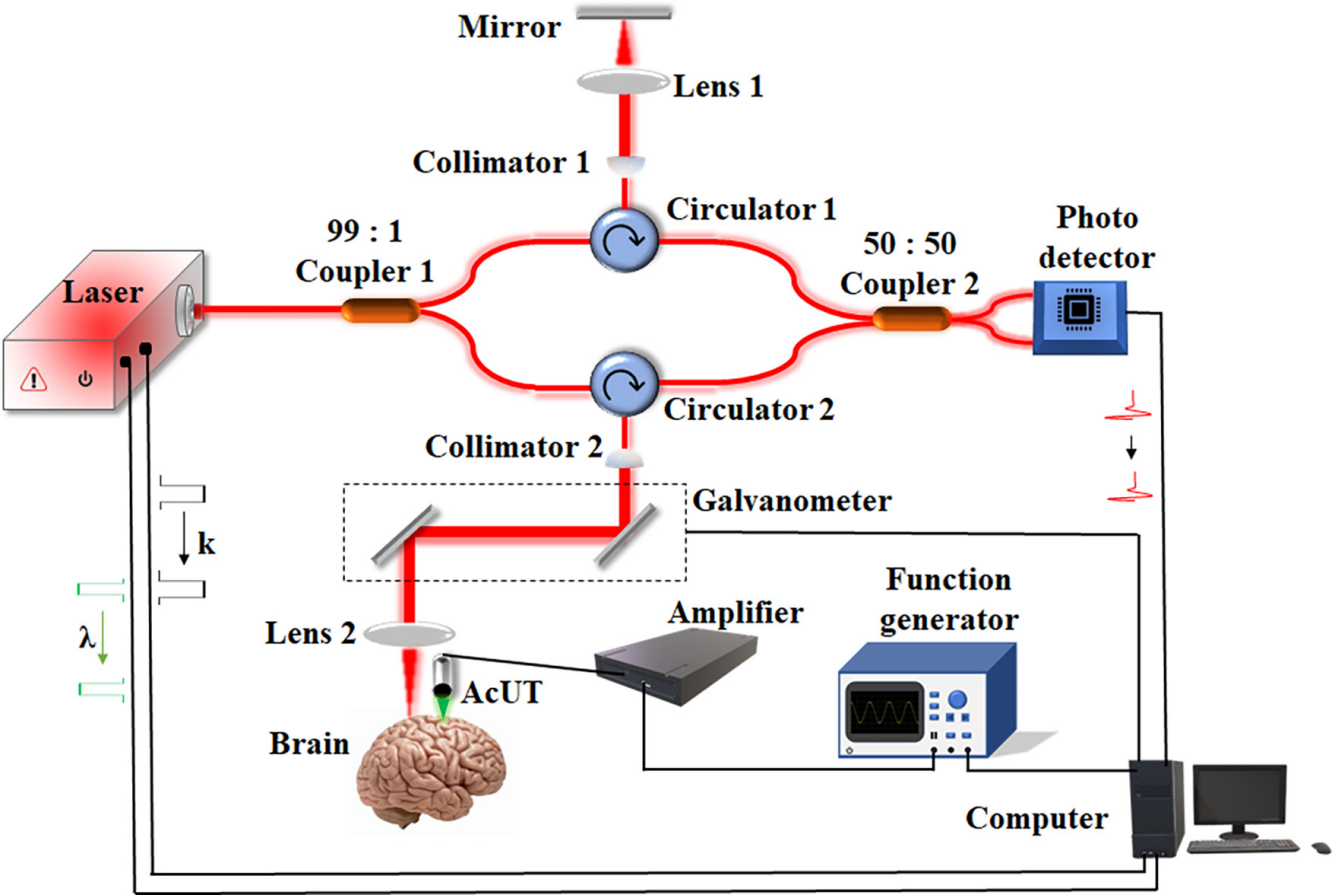
Schematic diagram of the AcUT-OCE system. A laser with a center wavelength of 1310 nm was divided into a sample arm (99%) and a reference arm (1%). The M-B scan protocol was completed by a two-dimensional (2D) galvanometer, which was driven by a computer. The λ trigger and k clock of the laser were used to perform synchronized control between the optical detection and ultrasound excitation systems.

To reduce the influence of the surface medium on the boundary conditions of brain tissue during SRW propagation, we designed and developed an AcUT-OCE system for noncontact imaging, and a schematic diagram of the system is shown in Fig. 8. A custom air-coupled ultrasound transducer is used for noncontact excitation of the SRW in the brain tissue, with a center frequency of 250 kHz, a focal length of 60 mm, and a focal spot diameter of 4 mm. The aluminum cylindrical housing of the AcUT, which had an outer diameter of 25 mm, is ground connected to provide an effective electromagnetic shield. The AcUT is connected to a bandwidth amplifier (ENI-2200L) via a BNC cable. A burst signal with a center frequency of 250 kHz is produced by a function generator (AFG31102) and used to drive the AcUT through a bandwidth amplifier. The clock signal is generated by the k-clock of the swept laser, which is synchronized with the sampled clock of the OCE interference spectrum. A home-build phase-sensitive OCT system combined with an M-B scan model is used to detect the SRW propagation process, as previously described.^57,58^ The OCT system had an A-line rate of 50 kHz, a center wavelength of 1310 nm, a scan range of 100 nm, an spatial resolution of 5.7 *μ*m (axial) × 15 *μ*m (lateral), an image depth of 5 mm (in air), a signal-to-noise ratio (SNR) of 105 dB, and a displacement sensitivity of 13 nm. In the M-B scan model, each M-scan consisted of 500 A-scans (a total of 10 ms), the B-scan consisted of 1000 M-scans along the lateral axis (a total of 10 s), and 512 sample points in depth will be completed. For each M-scan, from the 101st to 120th A-scans, a total of 20 ultrasonic pulses were generated (a total of 400 *μ*s) to excite SRWs in the sample. The size of the 3D dataset (x, z, t) is (1000, 512, and 500).

## SUPPLEMENTARY MATERIAL

See the supplementary material for details of 3D (x, z, t) reconstruction results of the SRW and SAW propagation processes in phantom (supplementary material video 1). The full videos of the 2D (x, z) and 3D (x, z, t) reconstructed SRW and SAW propagation processes in the FL region are shown in supplementary material videos 2 and 3, respectively. The full videos of the 2D and 3D reconstructed SRW and SAW propagation process in the PL region are shown in supplementary material videos 4 and 5.

## Data Availability

The data that support the finding of this study are available from the corresponding author upon reasonable request.
